# Damage Development on the Surface of Nickel Coating in the Initial Period of Erosion

**DOI:** 10.3390/ma14113123

**Published:** 2021-06-07

**Authors:** Dominika E. Zakrzewska, Marta H. Buszko, Alicja K. Krella, Anna Komenda, Grzegorz Mordarski, Robert P. Socha

**Affiliations:** 1 Research and Development Center of Technology for Industry, Ludwika Waryńskiego 3A, 00-645 Warszawa, Poland; dzakrzewska@imp.gda.pl (D.E.Z.); mbuszko@imp.gda.pl (M.H.B.); anna.komenda@cbrtp.pl (A.K.); 2 Institute of Fluid Flow Machinery PAS, Fiszera 14, 80-231 Gdańsk, Poland; 3 Jerzy Haber Institute of Catalysis and Surface Chemistry PAS, Niezapominajek 8, 30-239 Kraków, Poland; grzegorz.mordarski@ikifp.edu.pl; 4 Hanplast, Paciorkiewicza 3, 85-862 Bydgoszcz, Poland

**Keywords:** cavitation erosion, nickel coating, corrosion

## Abstract

The common occurrence of the phenomenon of cavitation in many industries and the multitude of factors affecting the resistance to cavitation erosion of used materials contribute to the search for methods and appropriate parameters of coating application that are able to minimize the effects of erosion. To determine the validity of the developed application parameters and the method used, cavitation studies and microscopic observations of the development of erosion during the cavitation test were carried out. There was a clear lack of incubation time and a linear increase in losses after 60 min of the test. Moreover, the damage observed during the test overlapped, widening the area of erosion and thus leading to damage to the integrity of the coating.

## 1. Introduction

One of the common causes of damage to turbines, marine propellers, pumps, or other components of hydraulic machinery, which contribute to their faster failure, is cavitation erosion [1,2,3]. Great interest in the phenomenon over many decades shows not only the scale of damage [4,5,6,7,8,9] but also the problem of compiling one correlation including all factors affecting the process of cavitation damage [10,11]. The cause of cavitation erosion is the phenomenon of cavitation, which is caused by the formation and collapse of bubbles in liquids that are subjected to frequent pressure changes [12]. Cavitation bubbles with the possibility of several times of growth and imploding arise from cavitation embryos, which are insoluble gases contained in the liquid [13].

The repeated interaction of micro-jet and shock wave resulting from the implosion of the cavitation bubble near the material causes the destruction of its surface. Degradation is mainly characterized by a change in property and geometry of the surface layer, due to the formation of microcracks that leads to mass loss [14]. The analysis of mass losses, erosive processes, and properties of the material subjected to erosion is used to select the criteria determining the resistance to cavitation erosion (CER) of materials and to develop materials, including coating materials with high cavitation erosion resistance. [15]. The development of resistance to cavitation erosion of the material is associated with the development of methods to increase it, such as heat treatment, chemical-heat treatment, and application of coating [8,16,17,18,19,20,21].

According to the studies of Hattori et al. [22,23], Franc [24], Man et al. [25], and Meng et al. [26], hardness is one of the main criteria affecting material resistance to cavitation erosion. Therefore, hard materials, including hard coatings often have good resistance to cavitation erosion [27,28]. However, as it has been shown in the studies performed by Taillon et al. [27], Krella [28] and Hong et al. [29], the high hardness does not always ensure good CER. Moreover, the value of hardness of the coating includes many factors, such as deposition parameters. Vespa et al. [30] have shown that the increasing input energy affected the decrease in hardness of WC/Ni-based coatings from 578 HV_10_ to 410 HV_10_. A similar observation has been obtained by Antoszczyszyn et al. [31], where, apart from the decrease in hardness of Ni-based coatings with an increase in the deposition current, the influence of the substrate on the hardness of the coating was proven. The influence of the deposition parameters on the hardness of Ni coatings has been proven by Lian et al. [32]. Moreover, a correlation between the deposition parameters and the hardness of the coating has been proposed. Although in most cases less wear was achieved with higher hardness, no relationship was found between wear resistance and hardness. Thus, besides hardness, other factors also influence erosion and wear resistance.

Other factors of the coating influencing the CER are good adhesion and corrosion resistance. The latter is especially important as the cavitation phenomenon takes place in a liquid. Among many coatings, nickel coatings are especially important due to their high hardness and good corrosion resistance. Investigations of nickel-based coatings confirmed its good cavitation erosion resistance [33,34,35,36]. However, the mechanical and strength properties of the coatings depend on the deposition method and parameters. Therefore, the test results obtained for coatings deposited by one method do not have to be representative for the same coatings deposited by another method. The electroless deposition of metallic layers allows for high hardness and large density of the coatings when compared to electrodeposited ones. The electroless method utilizes the reduction of nickel (II) cations by hypophosphite anions. The resulting Ni–P alloy can contain up to 14% of phosphorus [37].

The aim of the study was to investigate damage development on the surface of nickel coating as a result of cavitation erosion. Studies were carried out at short time intervals to analyze the effect of cavitation loads in time on coating erosion and at very low cavitation intensity.

## 2. Materials and Methods

### 2.1. Material

The samples used in cavitation tests were made of Nimax steel. The Nimax steel is used for the production of injection molds. The surface roughness parameters Ra and Rz before coating deposition were 0.34 ± 0.03 µm and 2.63 ± 0.29 µm, respectively. The surface roughness was measured in twelve different locations.

The chemical composition and mechanical properties are presented in Table 1 and Table 2, respectively.

A nickel coating is applied to deposit on the Nimax steel surface using the electroless deposition method. The solution for electroless deposition of the Ni–P films contained: (a) a source of Ni^+2^, NiSO·6H_2_O at 0.10 mol dm^−3^; (b) a reducing agent, NaH_2_PO_2_·H_2_O at 0.20 mol dm^−3^; and (c) a complexing and buffering solution of succinic acid (C_4_H_6_O_4_) and sodium succinate (Na_2_C_4_H_4_O_4_·6H_2_O) both at 0.20 mol dm^−3^. The solutions prepared in this manner showed pH quite close to 4.5. However, when needed, pH was kept lower with 1.0 mol dm^−3^ sulfuric acid or increased with 1.0 mol dm^−3^ ammonia to approach a pH of 4.5. The alloy coatings were deposited on the flat samples made of Nimax steel. Prior to the deposition, the pH of the deposition bath was measured, and the samples were cleaned with 1.0 M HCl to remove the thin layer of oxide. After triple rinsing with distilled water, the samples were added to 250 mL of the appropriate bath solution at 383–388 K. The beginning of deposition was noted by the evolution of hydrogen gas followed by the appearance of a silvery metallic coating on the sample surface. The samples were plated for 15 or 30 min after the first signs of deposition were noted. After the deposition was complete, a final pH reading was made and an average thickness for the deposition was calculated. The phosphorus concentration in the Ni–P coating was 8.0 ± 0.5 wt % as measured by X-ray fluorescence.

### 2.2. Electrochemical Test

**Figure 1:**
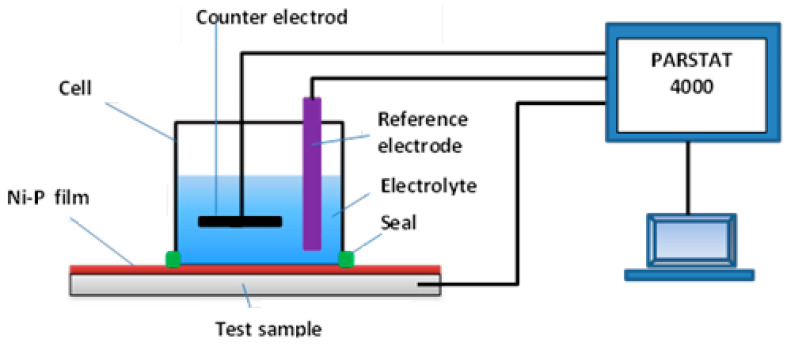
Scheme of the system for measuring the resting potential.

The electrochemical measurements of the nickel coatings were performed in 35 g dm^−3^ NaCl electrolyte. The solution was prepared from ppa chemicals. The electrochemical measurements were performed with the potentiostat galvanostat EIS Analyzer PARSTAT 4000 made by AMETEK (Oak Ridge, TN, USA). A dedicated cell working with three-electrode mode was used (Figure 1). A platinum foil was used as a counter electrode and an Ag|AgCl electrode in the saturated KCl solution was used as a reference electrode. The coated sample was used as a working electrode.

### 2.3. Cavitation Erosion Test

The cavitation tests were performed in a cavitation tunnel equipped with a system of barricades. The exact description of the tunnel as well as the description and diagram of the test stand is described in Ref. [38]. The test rig is equipped with instruments for measuring pressure at the inlet (*p*_1_) and outlet to the chamber (*p*_2_), and temperature of the fluid (T). The cavitation test was carried out using tap water as the working liquid with the following parameters: inlet pressure *p*_1_ = 400 ± 2 kPa, outlet pressure *p*_2_ = 120 ± 0.5 kPa, temperature T = 20 ± 1 °C.

The total time of the test was 120 min. The total test time was divided into time intervals of 5, 10, 20, 30, 45, 60, 80, 100, and 120 min. Before the test and after each exposure interval, the measurements of mass loss were made using a XA 160 analytical balance with accuracy class 1, measurement accuracy is 0.1 mg. Each mass loss measurement was performed five times. These measurements were used to derive the mean and standard deviation. Furthermore, after each exposure intervals, the microscopic observations and 2D and 3D profiles of surfaces were made on a Nikon Eclipse Ti-S optical microscope with a Nikon DS-Fi2 camera (Nikon Instruments Europe B. V., Amsterdam, The Netherlands). Furthermore, the microhardness measurements were carried out using the FALCON 401 Vickers Hardness (INNOVATEST, Shanghai, China) tester with 10 g load and dwell time 10 s. The load was selected so that the indentation depth was less than 1 µm, i.e., less than 1/10 of the coating thickness. Moreover, hardness measurements in a mode of continuous multiple cycling loading using an NHT³ nanoindentation tester (Anton Paar, Graz, Austria) were performed to learn the influence of cycling loading on the change of coating properties: hardness and elastic modulus. These measurements were performed at the acquisition rate of 20 [Hz] for 10 cycles, linear increase of the maximum load, the first load equal to 100 mN, unloading to 20.00%, maximum load equal to 350 mN, the time to reach the maximum load was 10 s, the time to unload was 10 s, dwell time was 3 s.

## 3. Results

### 3.1. Ni Coating

**Figure 2:**
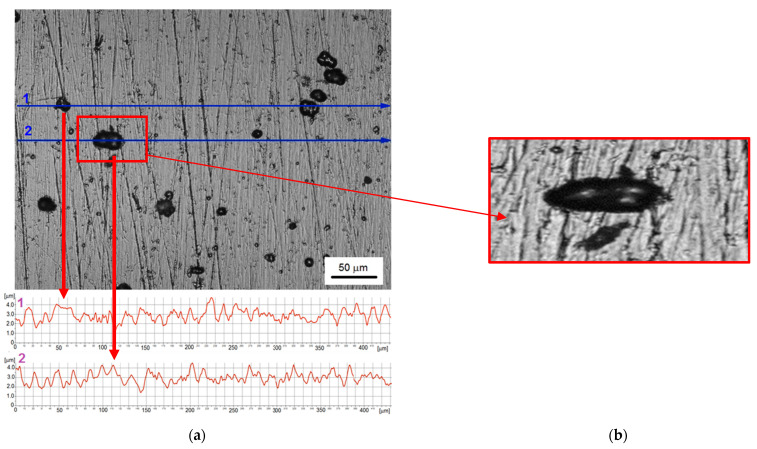
Nickel coating before cavitation erosion tests: (**a**) surface morphology with roughness profiles along the selected measurement lines; (**b**) 3D profile of a surface defect.

The Ni coating investigated in the presented studies is shown in Figure 2. The microscopic observation revealed the coating reflected the substrate very well and numerous defects with diameters ranging from 2 µm to even 46 µm were formed. The median size of defects was 9.6 µm the standard deviation was 8.7 µm. In addition, the roughness measurements showed that the surface unevenness had a height up to 2.5 μm and the defects were convex. Nevertheless, the coating was applied evenly to the substrate, and the observed unevenness was due to the unevenness of the substrate surface due to the low thickness of the nickel coating (about 10 μm).

Moreover, the measurements of the hardness of the substrate and the coating showed more than twice the hardness value of the nickel coating (1057 ± 13 HV) than the Nimax (426 ± 3) HV steel. The coating hardness is higher than the hardness of Ni–P coating (845 ± 7.6 HV) obtained Samanta et al. [39], although the same electroless method was used. In addition, the hardness of the produced electroless coating is much higher than that of Ni-electroplated coating (560 ± 12.8 HV) obtained by Samanta et al. [39]. Such a high hardness in comparison to the hardness of the Ni-based coatings produced by other methods [30,31,32] may prove the rightness of the selection of the coating application method to obtain high hardness.

**Figure 3:**
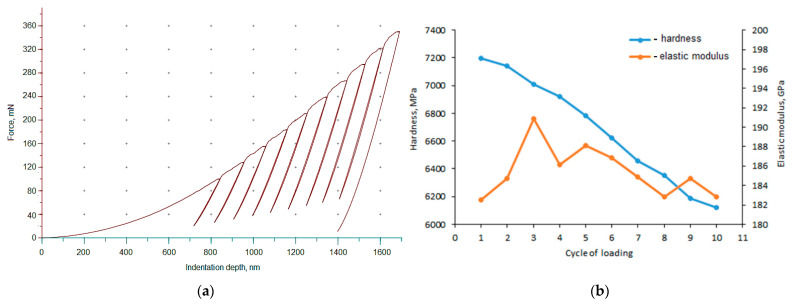
Force versus indentation depth curves for a Ni–P coating: (**a**) hardness and elastic modulus change with loading cycle (**b**).

The results of cycling loading are shown in Figure 3. The first load caused an indentation depth of about 0.8 µm, so it was less than 1/10 of coating thickness. With increasing cycles of load, the indentation depth increased as well. The maximum indentation depth during the third cycle was approx. 1.05 µm. Thus, starting from the third cycle, the indentation depth was higher than 1/10 of the coating thickness and the substrate began to have an influence on the results obtained. After all cycles, the maximum indentation depth was approx. 1.7 µm and the permanent indentation depth was 1.4 µm. With the increasing number of cycles, hardness decreased. After 10 cycles, hardness decreased over 1000 MPa. Thus, the softening effect occurred. In addition, the bigger drop of hardness arisen at the third cycle, which is related to the depth of indentation. However, the subsequent cycles resulted in a steady drop in hardness at a rate of 150 MPa per cycle. The modulus of elasticity increased during the first three cycles but then decreased. This study shows that the cyclic load significantly influenced the hardness and modulus of elasticity of the tested Ni–P coating and steel substrate, especially after the 3rd cycle, when the depth of the indentation is so deep that the substrate influences the deformation process of the entire coating-substrate system. Changes in hardness and modulus of elasticity made the coating more susceptible to plastic deformation.

### 3.2. Corrosion Resistance

**Figure 4:**
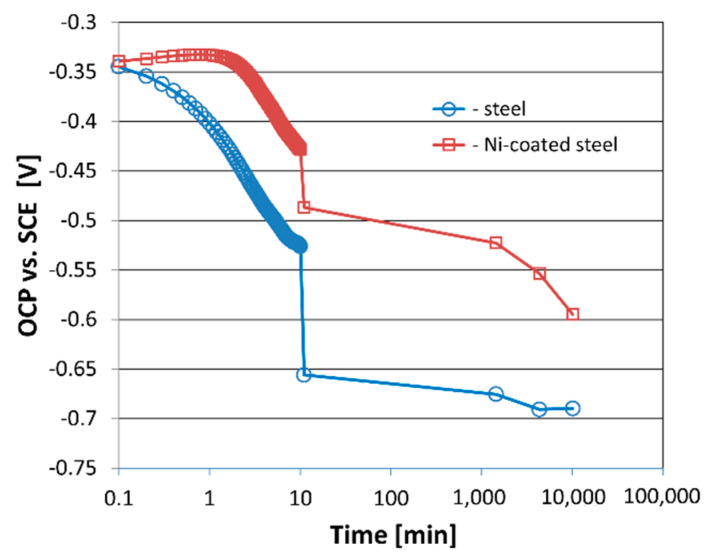
The dependence of the electrode potential versus the time after immersion to 3.5% NaCl solution.

The open circuit potential (OCP) value and its change within immersion time indicate resistivity of the coating against corrosion processes. The OCP data acquisition was started immediately after the introduction of the sample to the solution (c.a. 10 s after immersion of the sample). The measurements were recorded non-linearly. In the first 10 min, the measurement was performed every second. Then the period between the following measurements was extended to 1, 5, 24, 72, and 168 h, respectively. The OCP results are collected in Figure 4. The OCP measurements on Ni–P layer coated steel are compared to the Nimax steel substrate. The OCP value for Ni–P coated steel is found higher more than 100 mV when compared to the Nimax steel. Such behavior indicates corrosion protection of steel substrate by Ni–P coating. The observed slight increase of the OCP value for Ni–P coated steel at the very beginning of corrosion process can be assigned to the change in corrosion process at Ni–P surface caused by adsorption of [PO_4_]^3−^ ions as suggested by Lee et al. [40]. The observed lack of stabilization of the corrosion process for Ni–P coating after relatively long time of 10,000 min, can be a result of pits formation under the coating layer [40,41,42].

### 3.3. Cavitation Erosion Test

**Figure 5:**
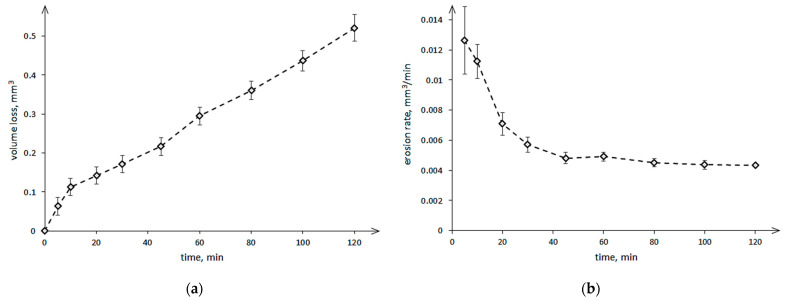
Results of the cavitation erosion of nickel coating: (**a**) volume loss in time and (**b**) cumulative erosion rates in time.

The results of the cavitation erosion of nickel coating are as a curve of volume loss in time (Figure 5a) and as a curve of cumulative erosion rates in time (Figure 5b).

The curve in Figure 5a shows a large increase in volume loss during the initial 10 min of the test, which is well reflected in the curve of the erosion rate in time (Figure 5b). After 5 min of the test, the erosion rate reached the highest value and was 0.0126 was mm^3^/min. There is no obvious incubation period. Subsequent measurements of the volume losses at 20, 30, and 45 min of the test showed a continuous, almost linear increase in the volume losses with decreasing erosion rate. Beginning from 40 min of the test, the erosion rate remained unchanged, indicating a stabilization value of 0.0045 ± 0.00026 mm^3^/min (Figure 5b). Stabilization of the erosion rate and a high increase in the volume losses of the nickel-based coating at the initial stage of the cavitation erosion test were also observed by Meng et al. [26] and Lampke et al. [33], although they have used a different test device—an ultrasonic test apparatus. Meng et al. [26] have obtained that a stabilization erosion rate was reached after about 50 min of testing, so after comparable test duration. Lampke et al. [33] have obtained a stabilization erosion rate only after 5 h of testing. The difference between these two tests was the test method used (sample position). Meng et al. [26] have used the direct method, while Lampke et al. [33]—the indirect method.

### 3.4. Microscopic Observation

**Figure 6:**
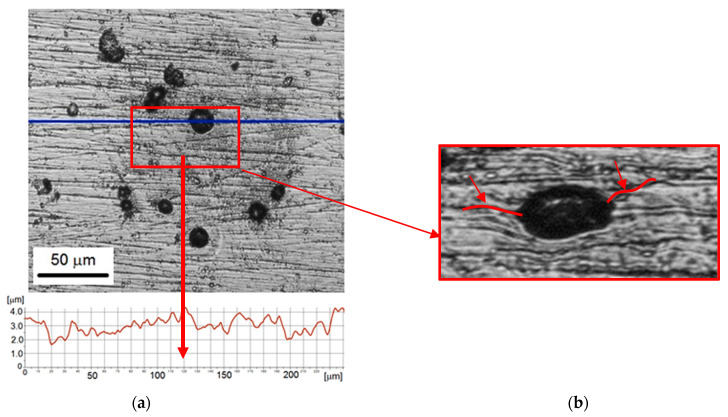
Nickel coating after 5 min of the cavitation erosion tests: (**a**) surface morphology with roughness profile along the selected measurement line; (**b**) 3D surface profile of a pit.

**Figure 7:**
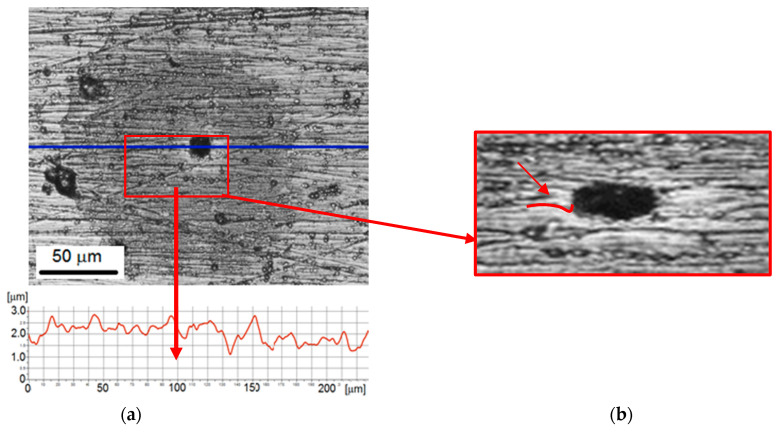
Nickel coating after 10 min of the cavitation erosion tests: (**a**) surface morphology with roughness profile along the selected measurement line; (**b**) 3D surface profile.

**Figure 8:**
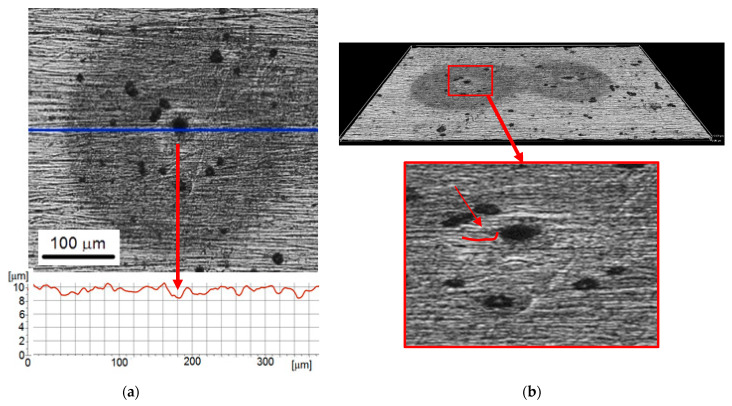
Nickel coating after 30 min of the cavitation erosion tests: (**a**) surface morphology with roughness profile along the selected measurement line; (**b**) 3D surface profile.

**Figure 9:**
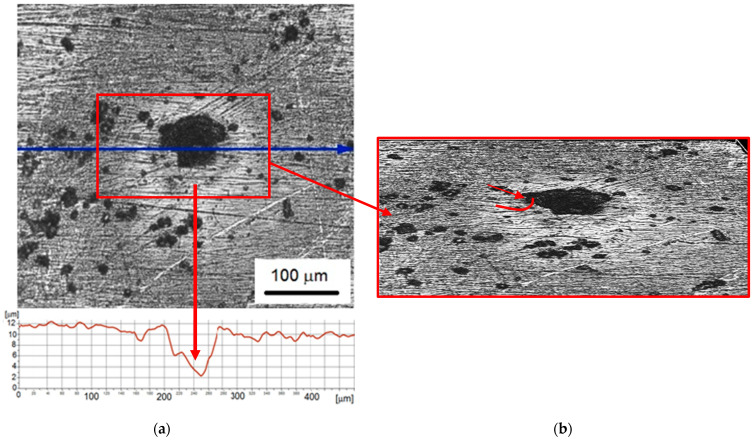
Nickel coating after 60 min of the cavitation erosion tests: (**a**) surface morphology with roughness profile along the selected measurement line; (**b**) 3D surface profile.

The development of Ni coating degradation starting from 5 min up to 60 min of the test was shown in Figure 6, Figure 7, Figure 8 and Figure 9. Observation performed after 5 min of testing revealed two types of damage formed on the coating surface: damage surrounded by circular shadings and small pits without such circular shadings. The pit surrounded by shadow is shown in Figure 6a. The diameter of this pit was 30 µm and the depth was about 1 µm. The 3D profile (Figure 6b) confirmed a concave shape of the edge of this pit (arrows). Other surface defects had a diameter of approximately 18–25 µm. The circular shadings had a diameter of about 150 µm and did not affect the 3D profile and the roughness profile, which was close to the roughness profile before cavitation tests. Very slight damage (pit depth) was likely an effect of the high hardness of the Ni coating.

Microscopic observations of the surface after 10 min of the cavitation test revealed circular shadings around the formed cavities on the observed surface (Figure 7). The round shading has become darker and more pronounced. The diameter of shaded circles around the pits was in a range from 200 µm to 400 µm, while the diameter of the pit from 20 µm to about 30 µm. Thus, the pit diameter was comparable to 5 min of testing. However, the edge of the pit was no longer concave but became convex (denoted by a red line and an arrow). The appearance of shading may be related to the oxidation of nickel (corrosion of nickel). According to research by Suslicek et al. [43], in the last stage of cavitation bubbles collapsing, the temperature inside the bubble exceeds 1000 K, and can even reach 5000 K. The high temperature and water environment in which cavitation tests are carried out meet the conditions necessary for the nickel oxidation process. The product of nickel oxidation is nickel oxide (NiO), which in the form of a thin film covers the eroded surface [44]. The performed observations showed that the appearance of shadows did not affect the 3D profile and surface roughness profile. The round shape of the shadows, in line with the shape of the imploding cavitation bubble, is also consistent with the assumption of nickel oxidation made. Thus, the observed shadows may be a thin layer of nickel oxide. On the other hand, it is known that the generated temperature inside the bubble drops sharply as a result of flowing cold water. It follows that only those cavitation bubbles that are in direct contact with the coating contribute to the oxidation of nickel and the formation of the observed dark shadows.

Extending the test duration to 30 min slightly affected the diameter of the shadows but had a significant effect on the change in the diameter of the pits (Figure 8). The diameter of shadows was about 300–400 µm, while the diameter of the pits was in the range from 20 to 40 µm. Thus, the diameter of the pits increased. In addition, Figure 8b shows that pits had a characteristic shape: the surface just around the pit was slightly convex, and next to it was concave. The roughness profile (Figure 8a) shows the pit depth of about 2 µm. As the possibilities of surface analysis with an optical profilometer are limited, the formation of a microcrack at the bottom of this pit cannot be excluded. This damage shape was likely the reason for corrosion development. Corrosion tests (c.f. Section 3.2) have shown lower corrosion resistance of the steel substrate in comparison to the Ni coating. The other factor influencing the development of corrosion was an increase in stress level in the coating. Elongation of the test duration means an increase in the number of impacting cavitation pulses. Although some of them are too weak to cause a pit in the hard nickel coating, they are able to supply some of the impact energy and stresses to the surface layer. The increase in stresses promotes the acceleration of corrosion processes.

With the increase of the exposure time to 60 min, the previously formed pits increased in diameter and depth (Figure 9a). The diameter of the pits ranged from about 35 µm to about 75 µm, the depth of the pit shown was 10 µm. Moreover, the diameter of the shadows around the pits increased even to 450 µm. The volcanic shape of the rim of the pit is even more visible—the edge of the pit became more and more convex. The conducted research shows that pitting corrosion accelerated with the increase of the duration of the cavitation erosion test. Pitting corrosion occurred in the form of pits initiated at the site of damage to the passive oxide layer. The damaged pits were the anode where the steel dissolves. Due to the small area of the anode, corrosion developed deeper into the tested steel. Moreover, the surroundings of the pitting were the cathode, and oxygen reduction could take place there. Corrosion pits can also negative effect by acting as stress risers. Fatigue and stress corrosion cracking may start at the base of corrosion pits [24,36,37].

## 4. Discussion

Electroless nickel-plating is widely used in anti-corrosion and wear engineering, but its application in CE protection has been rarely discussed in the literature. According to [45], the plated specimen sustained the lowest cumulative mass loss in a 3.5% NaCl solution. The corrosion tests presented in this article confirmed the protection of the steel substrate by the tested Ni–P coating. Hardness measurements showed a very high hardness of this coating, but cycling loads led to a decrease in hardness. The high hardness of the Ni–P coating also has a protective effect against cavitation erosion [12,16]. A much higher hardness than that obtained by Samanta et al. [39] may be due to a lower density of voids and pores, and/or lower grain size. Nevertheless, the differences in the corrosion resistance of the coating and the substrate, as well as the decrease in hardness and modulus of elasticity under cyclic loading conditions, affect the development of damage. Although the high hardness of the coating protects against cavitation degradation, a large difference in the hardness of the coating and the substrate can lead to accelerated erosion through delamination and removal of the coating [28,46,47].

Investigations of the electroless Ni–P coatings presented in [48,49,50] showed that the content of phosphorus affects porosity and corrosion resistance. The content of phosphorus depends on many parameters, such as concentration of the phosphorus donor in the bath, pH, temperature, or current density. For example, Ni–P coating with a pH of 4.5 content about 9 wt % of phosphorus [48]. Singh and Ghosh [50] showed that increasing the phosphorus content from 8% to 16% reduced the corrosion resistance. Li et al. [51] indicated that Ni–P coatings with a phosphorus content in the range of 8 wt % to 14 wt % are mainly amorphous and hydrophobic. These tests also showed that the corrosion rate of the Ni–P coating, in which the P content was 15.36 wt %, changed slightly with the increase of the immersion time, in contrast to the tested L360 steel. Thus, literature studies have shown that Ni–P coatings, regardless of the P content, are characterized by a better corrosion resistance (a lower corrosion rate) compared to steels. The corrosion tests presented in this paper are in accordance with the results obtained in [48,49,50,51,52] and confirm the better corrosion resistance of the Ni–P coating comparing to the Nimax substrate steel, regardless of the immersion time.

The microscopic observations of the coating before cavitation tests showed that the surface irregularities were reflected by the coating due to its small thickness. However, the height of surface unevenness decreased by about 0.8 µm: from 2.63 ± 0.29 µm to about 2 µm. In addition, defects with a wide range of diameters were formed during coating deposition. According to [49], defects in electroless Ni coatings are related to the phosphorus content and coating thickness. Thus, the cause of the numerous and large defects was likely the high content of phosphorus.

As mentioned, the aim of these studies was to analyze the pitting development in the Ni–P coating. As in the phenomenon of cavitation erosion, many pulses with a wide range of amplitudes hit the surface of the solid body, contributing to its degradation, the selection of the test conditions is very important. Too intense cavitation does not allow such an analysis because many accompanying phenomena overlap, and it becomes impossible to show some degradation effects. Therefore, very short exposure times and low inlet pressure were chosen. The low inlet pressure was to provide a small number of pulses capable of degrading the hard coating.

First damages and volume loss were recorded after 5 min of the cavitation erosion test, despite the high hardness of the coating and low inlet pressure. The lack of the incubation period can be related to the uneven surface of the coating and coating defects. High roughness and surface defects contribute to the faster development of cavitation erosion [28,47,53]. According to [28], surface defects accelerate the loss of volume in the initial stage of cavitation tests. However, studies in [47] have shown that the number of defects, not their size, has such an effect on initial surface degradation in cavitation tests. Moreover, after 5 min of testing, individual pits surrounded by circular shadows were formed. This is evidence of a small number of strong cavitation impulses in the flow. Thus, the selection of the test conditions was correct. The diameter of the pits and shadows reached 30 and 150 µm, respectively. The depth of the pits was up to 1 µm, and the edge of the pit had a concave shape. The concave shape was probably the result of compression by the micro cavitation structured. As described in Section 3.4, the shadows were likely the thin film of nickel oxide, which was the product of nickel oxidation (nickel corrosion), which is common in Ni–P coatings [54].

Extending the test duration to 10 min did not affect the pit diameter, but increased the shading diameter almost 3 times. Such a large increase in the diameter of the shading may be caused by the softening of the coating, as shown by the research on the influence of cyclic loading. Because of the decrease in the hardness of the coating, the coating deformed more due to the collapse of the bubble, which increased the contact surface with the coating. The effect of high temperature described before could likely lead to an acceleration of the oxidation process and the increase of the shading diameter.

Further extending the test duration to 60 min increased the pit diameter to 75 µm, the pit depth to 10 µm, and the diameter of the shadows to 450 µm. Thus, the diameter of the pits and round shadows still increased with the duration of the test. More than a twofold increase in the pit diameter (from 30 to 75 µm) indicates that the decrease in coating hardness was so great that the micro-jet was able to cause puncture and removal of the coating. This development of the degradation process was also influenced by the difference in the electronegativity of the coating and the substrate and the increase in a complex stress state, which favored galvanic corrosion. The steel becomes the anode and the coating becomes the cathode. The resulting galvanic currents concentrate on the anode with a small area, which accelerates the anodic dissolution reaction [55,56]. Due to the lower corrosion resistance of the substrate steel and the introduced state of stress, the degradation of the coating-substrate system developed mainly in the steel substrate.

The diameter of the shadow indicates that the maximum contact diameter of the bubbles at the moment of implosion is of the order of 450 µm. Taking into account the previously discussed changes in the properties of the coating-substrate system under the influence of cyclic loads, it can be concluded that the diameter of the bubbles that can cause surface damage is of the same order. Thus, the size of cavitation bubbles developed in the flow under selected test conditions (pressure at the inlet to the cavitation chamber 400 kPa, pressure at the outlet 120 kPa) is approximately 450 µm. The presented studies also showed that despite rapid cooling, the high temperature generated inside the cavitation bubble in the last stage of its collapse affects the surface of the exposed material and contributes to the process of its degradation.

## 5. Conclusions and Observations

The performed investigations allowed us to form the following conclusions:

- The electroless deposition method produces a hard nickel coating with good corrosion resistance. The OCP value for Ni–P coating was higher more than 100 mV comparing to Nimax steel. However, some defects with a diameter of 9 µm to 20 µm were formed on the coating surface.
- Cycling loading decreased hardness over 1000 MPa. The change of elastic modulus was related to the cycle number: initially increased, but later decreased. Changes in hardness and modulus of elasticity made the coating more susceptible to plastic deformation.
- The degradation of the coating-substrate system caused by the cavitation erosion phenomenon started at the highest rate, which then decreased to a stable value after 40 min of testing.
- The micro-jet impacts caused pitting, while the dark circular shadows were probably formed by oxidation of nickel due to direct contact of collapsing cavitation bubbles with the coating. The damage to the passive film caused accelerated cavitation and corrosion degradation of the coating and substrate. As the testing time increased from 5 min to 60 min, the diameter of the pits and round shadows increased threefold.
- Despite rapid cooling by flowing cold water, the high temperature generated inside the cavitation bubble during its implosion affects the surface of the exposed material and the process of its degradation.

## Figures and Tables

**Table 1:** Chemical composition of Nimax steel, wt %, steel standard.

C	Mn	Cr	Ni	Fe
0.1	2.5	3.0	1.0	93.4

**Table 2:** Mechanical properties of Nimax steel according to steel standard.

Yield Point [MPa]	Tensile Strength [MPa]	Elongation [%]	Compressive Strength [MPa]	Hardness [HV]
785	1265	11	1000	389

## Data Availability

Not applicable.
